# Buffer glucose adjustment affects myocardial function after ischemia–reperfusion in long‐term diabetic rat isolated hearts

**DOI:** 10.14814/phy2.15387

**Published:** 2022-11-02

**Authors:** Claudius Balzer, William J. Cleveland, Zhu Li, Matthias L. Riess

**Affiliations:** ^1^ Department of Anesthesiology Vanderbilt University Medical Center Nashville Tennessee USA; ^2^ Department of Anesthesiology University Medicine Greifswald Greifswald Germany; ^3^ Department of Anesthesiology TVHS VA Medical Center Nashville Tennessee USA; ^4^ Department of Pharmacology Vanderbilt University Nashville Tennessee USA

**Keywords:** contractile function, hyperglycemia, Langendorff, type 2 diabetes mellitus, ZSF1

## Abstract

Due to its comorbidities type 2 diabetes mellitus (T2DM) and hypertension, the Zucker Spontaneous Hypertensive Fatty (ZSF1) rat is a clinically relevant animal model when assessing ischemia–reperfusion (IR) injury. Most IR studies in hearts isolated from diabetic animals have been conducted at normal glucose concentrations, providing a different environment compared to in‐vivo. We hypothesized IR injury to be attenuated in isolated hearts of diabetic ZSF1 rats when adjusting the Krebs‐buffer (KB) to their in‐vivo, i.e., elevated blood glucose (BG) levels. Diabetic and non‐diabetic ZSF1 rats were anesthetized, hearts isolated and Langendorff‐prepared. While standard KB was used for the non‐diabetic and diabetic unadjusted groups, KB with glucose levels increased to each rat's prior BG level was used for the adjusted diabetic group. All hearts underwent 30 min ischemia and 120 min reperfusion. Diastolic contracture during ischemia and early reperfusion was delayed and temporarily attenuated in the adjusted compared to the unadjusted diabetic and the non‐diabetic groups. The decrease in coronary flow on reperfusion was attenuated in diabetic animals. Left ventricular developed pressure and contractility were not different among the three groups. Infarct size was significantly lower in non‐diabetic animals; buffer adjustment made no difference in diabetic animals. In our study, T2DM did not worsen myocardial function in ZSF1 rat isolated hearts. Since our results reveal that hearts with an adjusted glucose level exhibit an at least temporary improvement of function following IR, further studies should consider adapting glucose levels to create more realistic conditions in isolated, perfused hearts.

## Abstract 2

This study, for the first time, shows that adjusting buffer glucose concentrations in Langendorff‐prepared hearts from diabetic animals to better reflect prior in‐vivo levels led to a partial and temporary improvement in myocardial function. Importantly, overall myocardial function was not worse in diabetic compared to non‐diabetic animals. Only infarct size was lower in non‐diabetic animals, and was not affected by buffer adjustment in diabetic animals.

## 1 INTRODUCTION

An increasing number of patients suffering from cardiovascular diseases associated with type 2 diabetes mellitus (T2DM) and metabolic syndrome in the US and worldwide lends great importance to research aiming to improve outcomes (Khan et al., 2020). Patients with T2DM not only have a higher risk for cardiac arrest (Zaccardi et al., 2014), but also have a decreased chance of survival (Parry et al., 2017). Therefore, numerous basic research studies have investigated the negative effects of T2DM on heart function and increased myocardial susceptibility to ischemia–reperfusion (IR) injury in animal models. In particular, studies in Langendorff‐prepared hearts from rodents have shown long‐term T2DM to impair myocardial function. In addition, other animal models have been established, e.g., Zucker Diabetic Fatty (ZDF) or Zucker Spontaneous Hypertensive Fatty (ZSF1) rats, as clinically more relevant rodent models of obesity, insulin resistance and hypertension, respectively. By using Langendorff‐prepared hearts from ZDF rats, recent studies have shown decreased heart function and increased sensitivity to IR injury to underline the hypothesis of worsened myocardial function due to diabetes and metabolic syndrome (Hjortbak et al., 2018; Kristiansen et al., 2004; Pælestik et al., 2017; Povlsen et al., 2013). However, none of those studies has adjusted the Krebs buffer (KB) used for perfusing the isolated heart to the higher blood glucose (BG) levels in‐vivo.

The aim of the following study, therefore, was to investigate myocardial function and infarct size related to higher glucose levels by comparing a standard KB to a modified KB adjusted to the in‐vivo BG concentration immediately prior to heart isolation. We hypothesized that this adjustment positively affects myocardial function and infarct size in hearts from diabetic animals. Furthermore, to the best of our knowledge, we are the first to use ZSF1 rats, an advanced breed of ZDF rats, which includes hypertension in addition to features of metabolic syndrome, i.e., obesity, hyperlipidemia, insulin resistance and decreased glucose utilization.

## 2 MATERIALS AND METHODS

### 2.1 Animals

All procedures were approved by the Institutional Animal Care and Use Committee (IACUC) at Vanderbilt University Medical Center, Nashville, Tennessee and performed in compliance with the ARRIVE guidelines (Kilkenny et al., 2010). Six‐week old male obese ZSF1 rats (*n* = 10) and their lean, non‐diabetic littermates (*n* = 7) were obtained from Charles River Laboratories. Prior to experimentation, all rats were housed for at least 18 weeks in our institute's animal care facility and monitored weekly for BG (Accu‐Chek Aviva, Roche) and body weight. While non‐diabetic animals were maintained on standard chow diet, obese animals received a high fat diet (Chow #5008, Labdiet) to develop T2DM.

### 2.2 Langendorff heart isolation

On the day of the experiment, non‐fasted rats were anesthetized with an intraperitoneal injection of Pentobarbital (45 mg kg^−1^), BG levels were measured with an iSTAT handheld analyzer (VetScan i‐STAT® with GC8 cartridges, Abaxis), and 1000 IU heparin were administered intravenously. Oxygenated non‐recirculated KB containing (in mM) 148 Na^+^, 4.7 K^+^, 1.2 Mg^2+^, 1.6 Ca^2+^, 127 Cl^−^, 27.8 HCO_3_^−^, 1.2 H_2_PO_4_^−^, 1.2 SO_4_^2−^, 5.5 glucose, 2 pyruvate, 0.026 EDTA, and 5 U/L insulin was used for the non‐diabetic and the unadjusted diabetic groups; in the diabetic adjusted group, the KB glucose was increased to the BG levels of each individual animal by adding the adequate amount of glucose to the KB before the experiment.

After a negative response to a noxious stimulus, animals were euthanized by decapitation followed by thoracotomy. The aorta was cannulated distal to the aortic valve, the heart was perfused retrograde with adjusted versus unadjusted ice‐cold KB, both venae cavae were ligated, and the heart was rapidly placed into a Langendorff support system and perfused at a constant pressure of 70 mm Hg at 37°C, as described before (Riess et al., 2002; Salzman et al., 2017). The perfusate was equilibrated with ~95% O_2_ and ~ 5% CO_2_ to maintain a constant pH of 7.40, and filtered in‐line (5 μm pore size). Isovolumetric left ventricular pressure (LVP) was measured with a saline‐filled latex balloon (Radnoti LLC) inserted into the left ventricle. The diastolic LVP was initially adjusted to 10 mm Hg at baseline (bl) so that any subsequent pressure increases reflected diastolic contracture. Systolic, diastolic, and developed (systolic–diastolic) LVP, and its maximal first derivative (dLVP/dt_max_) as an index of ventricular contractility were calculated. Electrodes attached to the right atrial and ventricular walls monitored atrial and ventricular electrocardiograms to calculate the spontaneous heart rate (HR) and identify arrhythmias. The rate‐pressure product (RPP) as the product of developed LVP and HR was calculated to correct for HR‐dependent changes in developed pressure. Coronary flow (CF) was measured in‐line with an ultrasonic flowmeter (model T106X; Transonic Systems) and normalized to heart weight. All analog signals were digitized (USB‐6343, National Instruments) and recorded at 200 Hz (Labview Full Development System 2014, National Instruments) for later analysis.

### 2.3 Experimental protocol

All hearts were allowed to stabilize for 20 min. Hearts were then subjected to 30 min of global no‐flow ischemia before 120 min of reperfusion with continuous monitoring of LVP, HR, and CF followed by tissue harvest and ventricular infarct size assessment. If ventricular fibrillation occurred on reperfusion, a bolus of 100 μg lidocaine was immediately injected in the aortic cannula. All data were collected from hearts naturally in, or converted to, sinus rhythm.

### 2.4 Infarct size measurement

At the end of each experiment, hearts were removed, weighed and their atria discarded. Ventricles were cut into 2‐mm transverse sections using a heart matrix. Incubation with 1% 2,3,5‐triphenyltetrazolium chloride in 0.1 M KH_2_PO_4_ buffer (pH 7.4, 38°C) for 10 min stains viable tissue red by dehydrogenase enzymes present in viable cells, with infarcted areas remaining white (Riess et al., 2009). Both sides of each slice were digitally imaged on green background, and their infarcted areas measured automatically by planimetry using Image J 1.44i software (NIH), its ColorThreshold plugin and a custom‐developed, calibrated macro ensuring fast and operator‐independent measurements (Shidham et al., 2011). Infarcted areas were averaged on the basis of their weight to calculate the total ventricular infarction of each heart.

### 2.5 Osmolarity calculation

Osmolarity in plasma and Krebs‐solution was calculated based on the most accurate formula described by Fazekas et al. (Fazekas et al., 2012):

$$\begin{array}{c} \text{mosmol}\;l^{-1}=1.9\;x\;\left(\mathrm{Na}+K\right)+\left(\text{glucose}\;\left[\mathrm{mg}\;{\mathrm{dl}}^{-1}\right]/18\right)\\ \;+\left(\text{urea}\;\left[\mathrm{mg}\;{\mathrm{dl}}^{-1}\right]\;x\;0.19\right)+5\; \end{array}$$

Please note that adjustments have been made to allow the use of glucose and urea values in mg dl^−1^.

### 2.6 Statistical analysis

Unless otherwise indicated, all values are expressed as a mean ± standard error of the mean (SEM) of % bl. The Shapiro–Wilk test was used for all continuous data to test for normal distribution; not normally distributed data were treated as non‐parametric as were ranked data. Groups were compared by Kruskal‐Wallis tests for non‐parametric data and ANOVA for parametric data. If a difference among the three groups was found, post‐hoc comparisons were done by Dunn's or Student–Newman–Keuls test, respectively. Differences were considered statistically significant when *p* < 0.05 (two‐tailed). Significance symbols: *adjusted versus non‐diabetic, ^†^unadjusted versus non‐diabetic, ^#^adjusted versus unadjusted. Calculations and artwork were performed using Graphpad Prism (Prism 8, GraphPad Software).

## 3 RESULTS

Raw data are available to the interested reader upon reasonable request and in accordance with federal guidelines set forth by the funding agencies.

### 3.1 Baseline data

**FIGURE 1:**
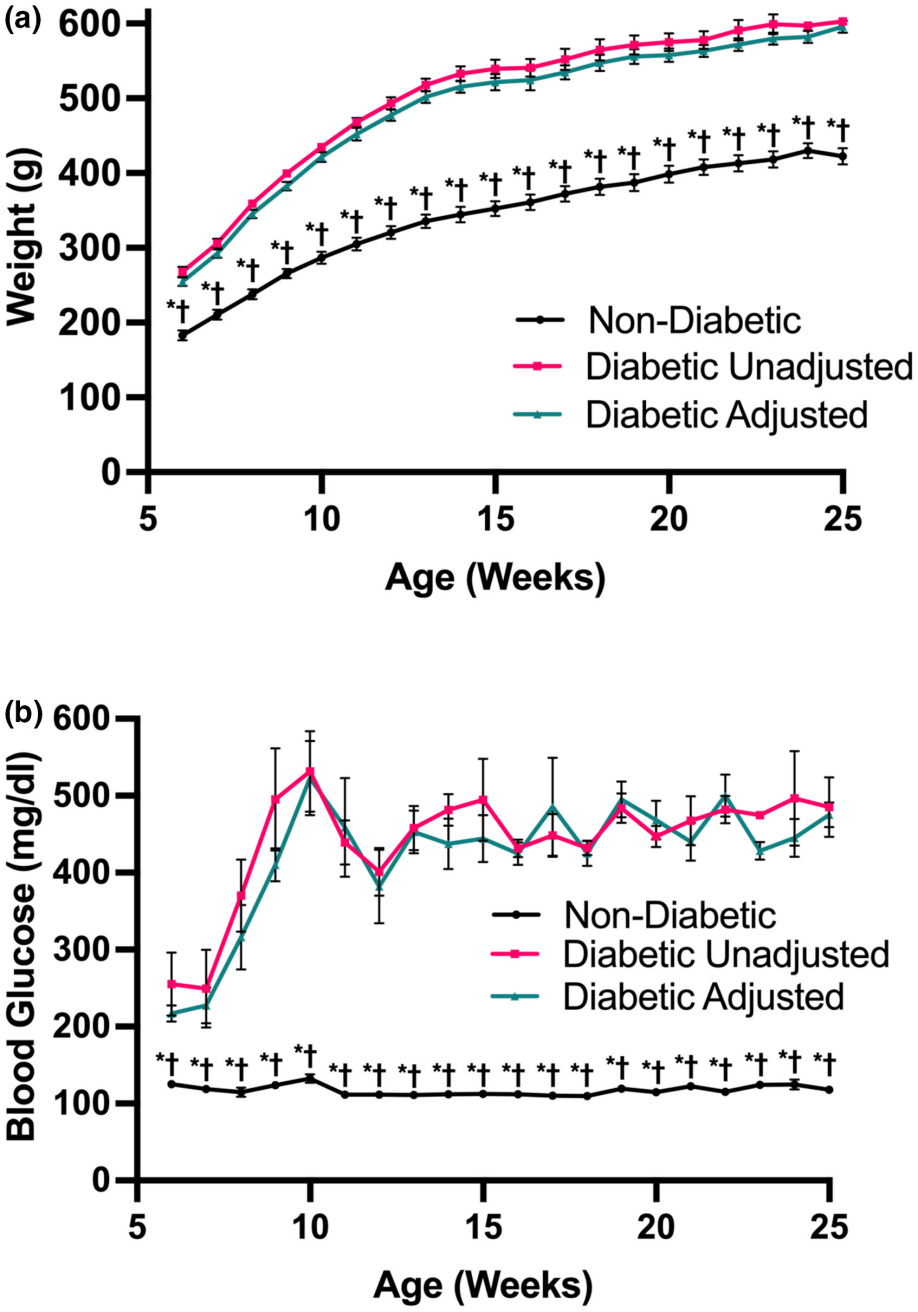
Development of weight (panel a) and blood glucose (panel B) over 25 weeks of lifetime before Langendorff experiments. Non‐diabetic (*n* = 7), diabetic unadjusted (*n* = 5) and adjusted (*n* = 5). All values are mean ± standard error of the mean (SEM). Statistics: Kruskal‐Wallis with Dunn's, *p* < 0.05 *adjusted diabetic versus non‐diabetic, ^†^unadjusted diabetic versus non‐diabetic. Please note that the SEMs of the non‐diabetic group are at times smaller than the symbols and therefore not visible.

There was no significant difference in age among the three groups (Table 1). Diabetic ZSF1 rats were significantly heavier than their non‐diabetic littermates at all times during housing in our animal facility (Figure 1a). Weekly BG measurements showed significant differences between diabetic and non‐diabetic animals, but no differences between the two diabetic experimental groups (Figure 1b). Similar results were seen on the day of the experiments (Table 1), with the exception of an unexplained statistical difference in BG values and heart weight between the two diabetic groups on the day of the experiment.

**TABLE 1:** Baseline values and heart weights

	Non‐diabetic (*n* = 7)	Diabetic unadjusted (*n* = 5)	Diabetic adjusted (*n* = 5)
Age (weeks)	26.3 ± 0.9	24.5 ± 1.1	27.2 ± 0.4
Weight (g)	454.9 ± 9.2	613.8 ± 13.5^†^	608.6 ± 13.1*
Heart Weight (g)	1.9 ± 0.1	2.1 ± 0.1	2.4 ± 0.1*
Heart Weight per 100 g body weight	0.42 ± 0.01	0.35 ± 0.02^†^	0.40 ± 0.02
Blood Glucose (mg dl^−1^)	115.0 (107.0, 129.0)	399.0 (332.0, 424.0)^†^	289.0 (264.5, 362.0)*^,#^
Buffer Glucose (mg dl^−1^)	96.0 (91.0, 100.0)	98.0 (93.0, 99.5)	280.0 (265.0, 348.5)*^,#^
Blood Osmolarity (mOsm)	290 ± 0.3	288 ± 1.1	284 ± 1.2*^,#^
Buffer Osmolarity (mOsm)	307 ± 1.1	309 ± 1.8	312 ± 4.5

*Note*: All parametric values are mean ± standard error of the mean, non‐parametric values are median (25th, 75th quartile). Statistics: Parametric: ANOVA with SNK, non‐parametric: Kruskal‐Wallis with Dunn's, *p* < 0.05 for *adjusted diabetic versus non‐diabetic, ^†^unadjusted diabetic versus non‐diabetic, ^#^adjusted versus unadjusted diabetic.

### 3.2 Myocardial and coronary function

**FIGURE 2:**
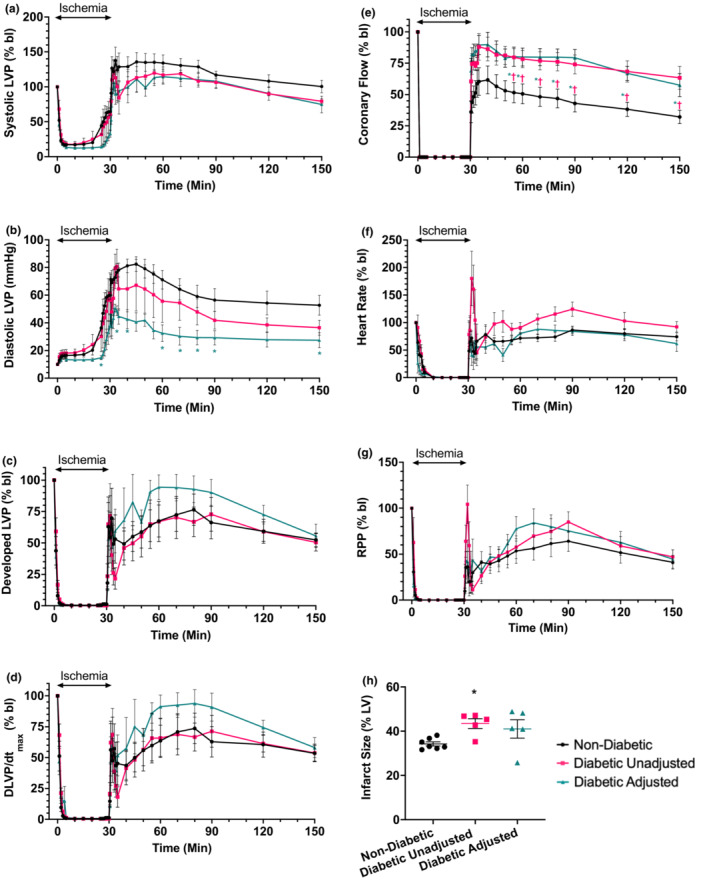
Time courses of (a) systolic (in % baseline, bl), (b) diastolic (in mm Hg), and (c) developed (systolic–diastolic, in % bl) left ventricular pressure (LVP), (d) dLVP/dt_max_ (in % bl), (e) coronary flow (CF, in % bl), (f) heart rate (HR, in % bl), and (g) the rate‐pressure‐product (RPP, in % bl) before, during, and after 30 min of global no‐flow ischemia in hearts isolated from ZSF1 rats. Panel H shows ventricular infarct size (in %) for each of the three groups. Non‐diabetic (*n* = 7), diabetic unadjusted (*n* = 5) and diabetic adjusted (*n* = 5). All values are mean ± standard error of the mean. Statistics: Kruskal‐Wallis with Dunn's *p* < 0.05 *adjusted diabetic versus non‐diabetic, † unadjusted diabetic versus non‐diabetic.

Systolic LVP, expressed as % bl, showed no significant difference in non‐diabetic ZSF1 rats compared to either of the two diabetic groups (Figure 2a). Diastolic contracture, expressed in mm Hg (Figure 2b), occurred in all hearts, starting during ischemia and lasting throughout reperfusion. While there was no difference between non‐diabetic and diabetic unadjusted experiments, diabetic hearts with adjusted glucose exhibited a significantly later increase in diastolic LVP during ischemia, and a significantly lower contracture both during ischemia as well as during early reperfusion compared to the other two groups. All of the above contributed to statistically not significant trends towards higher developed LVP (Figure 2c) and dLVP/dt_max_ (Figure 2d) for the adjusted diabetic hearts compared to the other two groups. CF (Figure 2e) returned at a significantly higher rate in both diabetic groups compared to hearts of non‐diabetic animals; there was no difference between the two diabetic groups. HR (Figure 2f) as well RPP (Figure 2g) showed no significant difference among the three groups.

### 3.3 Ventricular infarct size

**FIGURE 3:**
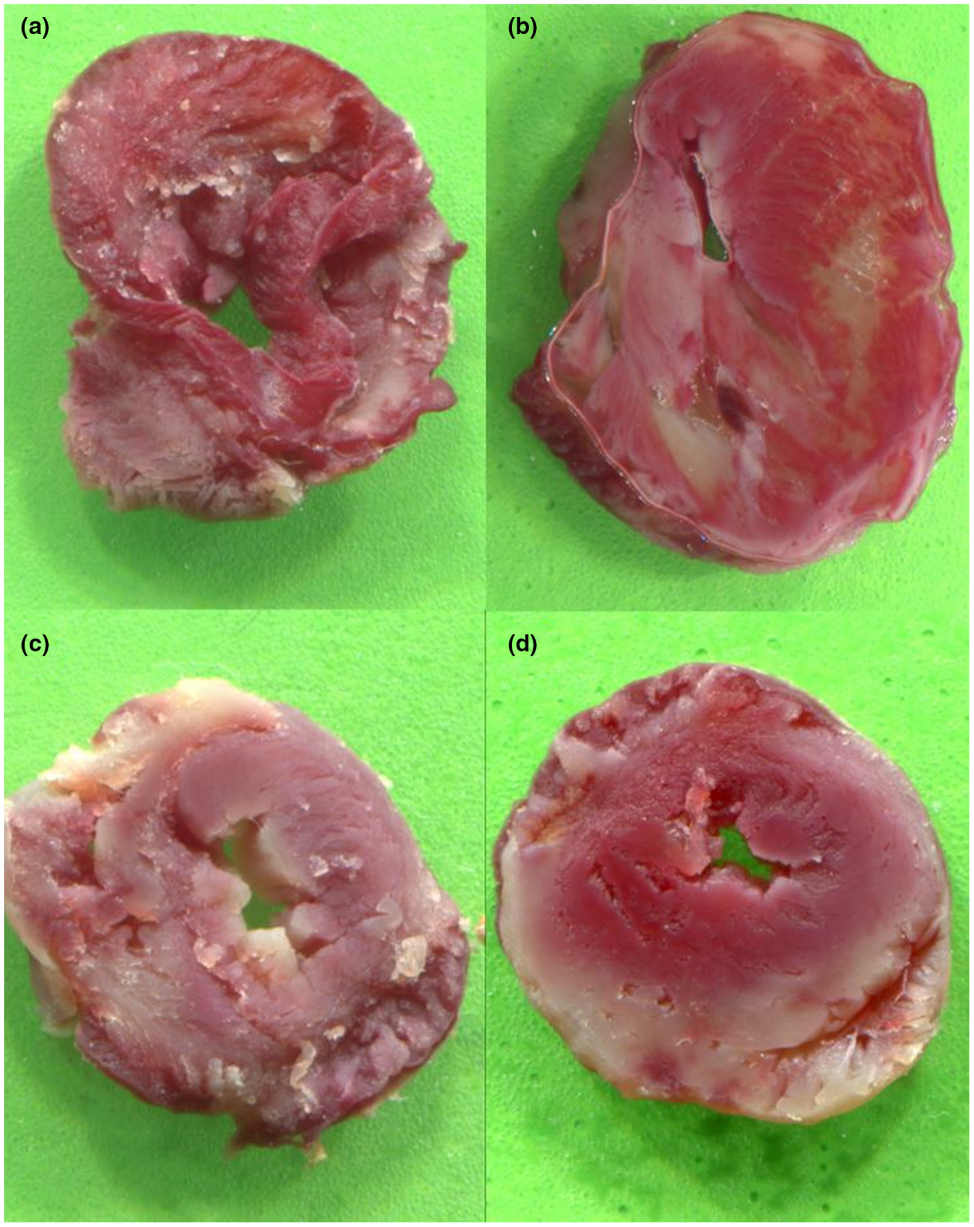
Infarct size scans of representative 2,3,5‐triphenyltetrazolium chloride‐stained slices of one of the diabetic unadjusted group hearts (a, b) and two slices of one of the non‐diabetic group hearts (c, d). While viable cells are stained red by dehydrogenases, infarcted areas remain white.

In contrast to the functional outcomes, infarct sizes of diabetic animals (unadjusted 43.5 ± 2.2 %LV, adjusted 41.0 ± 4.2 %LV) were higher than those from non‐diabetic rats (34.3 ± 0.9 %LV) (Figure 2h); glucose adjustment made no difference. Representative TTC‐stained slices are shown in Figure 3.

## 4 DISCUSSION

This study, for the first time, characterizes and compares the effects of adjusted higher glucose levels in KB related to myocardial IR in isolated hearts of diabetic and non‐diabetic ZSF1 rats and reports a partial and temporary improvement in hearts with adjusted higher KB glucose levels compared to lower levels in a standard unadjusted KB.

Diastolic contracture in diabetic ZSF1 rat hearts during ischemia and on reperfusion was temporarily improved compared to non‐diabetic ZSF1 rats. Under these conditions, T2DM had no negative impact on IR injury in our ex‐vivo model related to functional measurements, while infarct size was significantly higher in diabetic animals. In contrast, CF upon reperfusion was surprisingly better in both diabetic groups compared to non‐diabetic hearts; while we do not have a reasonable explanation for this observation, it shows that improved flow alone does not necessarily guarantee better function or less infarction.

Since this is the first study examining isolated hearts of ZSF1 rats related to IR, our results need to be compared to previous ZDF rat studies as ZSF1 rats are a cross between a ZDF female and an SHHF male rat (Bilan et al., 2011; Griffin et al., 2007). First, we did not detect preischemic differences between diabetic and non‐diabetic animals related to heart function by adjusting KB glucose in contrast to other ZDF rat studies (Burgdorf et al., 2006; Hjortbak et al., 2018). Furthermore, Wang et al. conducted a similar study with ZDF rats, showing the same improved function upon reperfusion in long‐term diabetic rats, while heart injury markers such as troponin increased during reperfusion in diabetic rats (Wang & Chatham, 2004). This is congruent to our finding of an increased infarct size in diabetic animals and was also seen after chronic diabetes in ZDF rats in‐vivo (Hoshida et al., 2000). However, Wang et al. did not adjust the KB glucose and reported a significant higher diastolic contracture in diabetic hearts during ischemia, while our study revealed a delayed and attenuated contracture in adjusted diabetic experiments compared to unadjusted KB. Wang et al. also stated that the substrate delivery during ischemia is the primary determinant of glycolytic flux, regardless of impaired glucose utilization due to diabetes. Because we provided higher glucose concentration before ischemia and because increased glycolysis is known to prevent contracture (Cross et al., 1996), this might have contributed to the observed delayed contracture in the diabetic adjusted group. However, ischemic contracture alone is not a reliable indicator for tissue injury (Kolocassides, Galinanes, & Hearse, 1996; Kolocassides, Seymour, et al., 1996), and we have not seen a difference in infarct size between the diabetic adjusted and unadjusted groups.

Although recommended for Langendorff experiments studying diabetes and hyperglycemia (Bell et al., 2011), we are one of a few groups who use a KB with glucose concentrations around 100 mg dl^−1^ in our controls to provide physiological condition to the rat hearts, while most Langendorff studies have used significantly higher KB glucose levels of 200 mg dl^−1^ and higher (Burgdorf et al., 2006; Chen et al., 2006; Hjortbak et al., 2018; Jilkina et al., 2003; Kristiansen et al., 2004; MahalakshmiA, 2018; Povlsen et al., 2013; Pælestik et al., 2017; Zálešák et al., 2016). In particular, those studies did also not adjust their KB for an even higher BG present in diabetic animals before the heart was isolated, and they reported a worsened outcome related to heart function and diabetes. Despite the fact that we were able to show the same worsened effect of long‐term diabetes related to infarct size, Povlsen et al. found that functional outcomes were impaired in isolated diabetic ZDF rat hearts when not adjusting KB glucose (Povlsen et al., 2013), while our study revealed a temporarily improved heart function by adjusted KB glucose levels.

Another possible factor influencing myocardial function after IR injury has been described by changing osmolarity of the perfusate. For example, Chen et al. were able to show an enhanced tolerance to ischemia in their model of a rodent type 1 diabetes with hyperosmotic perfusion in isolated hearts (Chen et al., 2006). Our study demonstrated just minor differences between diabetic and non‐diabetic animals related to osmolarity in‐vivo. More importantly, we also found only small deviations in osmolarity after adjusting the KB glucose in each group. Furthermore, the differences related to the environment in‐vivo and ex‐vivo were less than 10% in change of osmolarity and the influence of the missing oncotic pressure in the Krebs‐solution was also not taken into account. Furthermore, we chose to use insulin in contrast to other studies, decreasing the osmotic effect by glucose uptake of the cells (Wang & Chatham, 2004). Therefore, we expect that our findings related to myocardial function are based on a difference of individual glucose levels more than of osmolarity. The latter was calculated in our study and might not reflect the actual osmolarity, but the formula we used has been tested and described as one of the most accurate (Fazekas et al., 2012).

Our study needs to be interpreted within its natural constraints. Due to the simulated and simplified environment of the Langendorff setup, experiments only allow limited relevance with regard to the very complex influencing factors in‐vivo that could interfere with the heart's sensitivity towards IR injury. In this case, where ZSF1 rats mimic the metabolic syndrome in humans with a variety of clinically relevant comorbidities, we not only used an isolated heart model devoid of autonomic and hormonal input, but also a blood‐ and plasma‐free perfusate. With glucose as the sole substrate for myocardial metabolism, the KB does not provide all in‐vivo energy substrates, e.g., fatty acids. Therefore, it is possible that an increase of BG levels improves the energy status of rat hearts in the absence of fatty acids. However, none of the listed studies has used fatty acids in their KB. The addition of fatty acids to a Langendorff isolated heart setup is challenging due to the need to use bovine serum albumin with subsequent foaming and clogging as tested in our and other laboratories (Bell et al., 2011).

Since we are the first to study isolated hearts of ZSF1 rats, our study has the character of a pilot study. Our focus was on male ZSF1 rats, although female ZSF1 rats are also available, but do not develop hyperglycemia (Nguyen et al., 2020). Moreover, diabetic ZSF1 are not only a model of T2DM and metabolic syndrome, but also a well‐known rat model of heart failure with preserved ejection fraction and hypertension (Davila et al., 2019; Hohendanner et al., 2018), which may affect cardiac function. Unfortunately, and although the animals were randomly selected, we saw unexpected differences in heart weight in both diabetic groups that could have affected the outcome. Furthermore, diabetic rats showed a high variability of BG levels and we were unable to establish a useful control group with hyperglycemia in lean ZSF1 rat isolated hearts. The unexpected difference in BG levels on the day of surgery (Table 1) did not reflect the long‐term BG levels, but underscores that, for future experiments, the adjustment of glucose values in the Krebs buffer should ideally not be based on the value directly before the experiment but on the average value of the last week.

Because of the limited number of diabetic ZSF rats available for this project, a formal power and sample size analysis were not conducted, and a type II error cannot be safely ruled out. However, this pilot study of 17 rats will serve as basis for future studies with expanded biochemical analyses to investigate mechanisms of our findings.

## 5 CONCLUSION

This study, for the first time, shows that adjusting buffer glucose concentrations in Langendorff‐prepared hearts from diabetic animals to better reflect prior in‐vivo levels led to a partial and temporary improvement in diastolic myocardial function. Importantly, overall myocardial function was not worse in diabetic compared to non‐diabetic animals. Only infarct size was lower in non‐diabetic animals, and was not affected by buffer adjustment in diabetic animals. Further studies should consider adapting the glucose level to create more realistic conditions in isolated‐perfused hearts.

## CONFLICT OF INTEREST

No conflicts of interest, financial or otherwise, are declared by the authors.
